# Driving pressure vs. oxygenation-based PEEP titration strategies in ARDS patients: a physiological study

**DOI:** 10.1186/s13054-025-05459-8

**Published:** 2025-07-08

**Authors:** Silvia Coppola, Giulia Catozzi, Tommaso Pozzi, Alessandro Monte, Geraldina Besana, Laura Depedri, Davide Chiumello

**Affiliations:** 1 Department of Anesthesia and Intensive Care, ASST Santi Paolo e Carlo, San Paolo University Hospital, Via Di Rudini 9, Milan, Italy; 2 Department of Health Sciences, University of Milan, Milan, Italy; 3 Coordinated Research Center on Respiratory Failure, University of Milan, Milan, Italy

**Keywords:** ARDS, PEEP titration, Respiratory mechanics, Gas exchange

## Abstract

**Background:**

The aim of this study was to evaluate the effects of three different PEEP titration strategies in ARDS patients, one based on driving pressure and two based on oxygenation (one PEEP level according to the high PEEP/FiO_2_ table and a two fixed PEEP levels, i.e., 5 and 15 cmH_2_O, according to the modified Berlin definition severity), on gas exchange, partitioned respiratory mechanics and mechanical power during protective lung ventilation.

**Methods:**

Prospective observational study including 35 sedated, paralysed and mechanically ventilated patients with ARDS according to the Berlin definition within 48 h from admission to a medical-surgical ICU. Each patient underwent PEEP titration according to a clinical (based on driving pressure), empirical (based on high PEEP/FiO_2_ table) and fixed (5 or 15 cmH_2_O for patients with PaO_2_/FiO_2_ less than or greater than 150 mmHg) strategy. After 20 min of each phase, partitioned respiratory mechanics, gas exchange and hemodynamics were measured.

**Results:**

In the whole population, when comparing empirical *versus* clinical PEEP (15 [10–18] vs. 10 [8–10] cmH_2_O), end-inspiratory airway pressure, lung and respiratory system elastances, mechanical power, lung stress and PaCO_2_ were significantly higher, but PaO_2_/FiO_2_ was higher with empirical PEEP. In mild-moderate ARDS patients, end-inspiratory airway pressure and lung stress were significantly higher with clinical (8 [8–10] cmH_2_O) and empirical PEEP (10 [8–12] cmH_2_O) as compared to fixed PEEP (5 cmH_2_O); mechanical power was higher with empirical PEEP as compared to other PEEP titration strategies. Gas exchange did not differ. In moderate-severe ARDS patients, end-inspiratory airway pressure, lung stress, mechanical power, PaO_2_/FiO_2_ and PaCO_2_ were significantly higher with fixed (15 cmH_2_O) and empirical PEEP (17 [14–18] cmH_2_O) as compared with clinical PEEP (10 [8–10] cmH_2_O).

**Conclusions:**

Clinical PEEP titration provided better respiratory mechanics in terms of lower end-inspiratory airway pressure, lung stress and elastance and lower PaCO_2_ compared with a high PEEP/FiO_2_ table. In mild-moderate ARDS patients, a fixed PEEP level (5 cmH_2_O) provided lower end-inspiratory airway pressure and lung stress without detrimental effects on gas exchange compared to empirical or clinical PEEP. In moderate-severe ARDS patients, empirical and fixed PEEP (15 cmH_2_O) levels resulted in higher levels of end-inspiratory airway pressure, lung stress, PaO_2_/FiO_2_ and PaCO_2_ compared to more moderate PEEP levels.

**Supplementary Information:**

The online version contains supplementary material available at 10.1186/s13054-025-05459-8.

## Background

Patients with acute respiratory distress syndrome (ARDS) are characterised by hypoxemia, increased alveolar collapse, lung heterogeneity and small airway closure [1–3]. Protective lung ventilation in ARDS should provide a low tidal volume with positive end-expiratory pressure (PEEP) to recruit the lung and reduce the heterogeneity by counteracting the gravity-dependent alveolar collapse while improving gas exchange [3–5]. Since the first descriptions of its use, mainly to improve oxygenation and reduce the need for high inspiratory oxygen fractions, PEEP is currently used to promote lung recruitment, decrease mechanical heterogeneity and ventilator-induced lung injury (VILI) [6, 7]. Thus, PEEP should recruit collapsed lung regions and reduce intrapulmonary shunt [2, 4, 8]. However, in patients with poor lung recruitment, PEEP may increase stress and strain and promote VILI [8, 9]. Other adverse effects of PEEP include decreased venous return, increased pulmonary vascular resistance, decreased cardiac output and acute kidney injury [10–12].

Nowadays, PEEP setting is a controversial part of lung protective ventilation strategy and clinicians should balance its positive and negative effects.

Several strategies have been proposed to titrate PEEP during lung protective ventilation [13–15]. The most common strategies are based on PEEP/FiO_2_ tables, respiratory mechanics (respiratory elastance), end-expiratory transpulmonary pressure and PEEP trials at low and high PEEP levels [4, 16].

Three multicentre randomised clinical trials comparing the effects of a high *versus* low PEEP strategy according to respiratory mechanics or empirical PEEP/FiO_2_ tables found no difference in terms of outcome [17–19]. Subsequent randomised trials comparing PEEP selection according to esophageal pressure or PEEP/FiO_2_ strategy showed no difference in outcome [13, 20]. A subsequent meta-analysis of individual data from randomised trials showed that a high PEEP (≥ 15 cmH_2_O) strategy reduced hospital mortality by 5% in patients with severe ARDS [16]. However, in the recent ART randomised trial, a higher incidence of barotrauma, hemodynamic instability and mortality was observed with high PEEP (> 15 cmH_2_O) and aggressive recruitment maneuvers compared with a low PEEP strategy according to the low PEEP-FiO_2_ table of ARDS Network in moderate to severe ARDS [21]. A recent meta-analysis of 18 randomized controlled trials (RCTs) reported better outcomes with high levels of PEEP in patients with moderate to severe ARDS [22]. Similar to the findings of the ART trial, potential adverse effects of high PEEP levels include circulatory depression and lung overdistension, which may outweigh these beneficial effects [22, 23]. In addition, due to the heterogeneity and degree of disease that varies among ARDS patients, similar PEEP levels may have opposite effects (i.e., a more recruitment versus more overdistension and hemodynamic impairment) [6].

Recent guidelines on ARDS have been unable to recommend for or against PEEP titration according to respiratory mechanics, esophageal pressure or PEEP/FiO_2_ tables [3, 13]. However, all these methods mainly addressed only one side of the possible determinants of VILI such as driving pressure, end-inspiratory airway pressure or end-inspiratory transpulmonary pressure. To overcome the individual role of each component of mechanical ventilation, mechanical power has been proposed as a unifying parameter and main determinant to monitor VILI [24–26]. Previous data have shown that both on ICU admission and during the intensive care, the higher the mechanical power, the higher the mortality, with a possible safe threshold around 17 J/min [26–30]. Reducing mechanical power has been shown to improve short and long-term outcomes [31–33]. In an experimental healthy animal model, increasing PEEP over 11 cmH_2_O has been shown to result in a proportional increase in mechanical power, with associated higher fluid and vasopressor requirements and worse outcome [34]. However, there is a paucity of data on the individual choice of PEEP in ARDS patients and on the effect of mechanical power [14, 35–37].

The aim of this study was to evaluate the effects of three different PEEP titration strategies in ARDS patients, one based on driving pressure and two based on oxygenation, in terms of gas exchange, partitioned respiratory mechanics and mechanical power during protective lung ventilation. Three strategies were evaluated: in the whole study population (1) a clinical PEEP level based on driving pressure and (2) an empirical PEEP level according to the high PEEP/FiO_2_ table [13]; in addition, (3) two fixed PEEP levels (5 and 15 cmH_2_O) were applied and tested according to the severity of ARDS (PEEP 5 cmH_2_O when 150 mmHg < PaO_2_/FiO_2_ ≤ 300 mmHg and PEEP 15 cmH_2_O when PaO_2_/FiO_2_ ≤ 150 mmHg).

## Materials and methods

### Study population

**Fig. 1:**
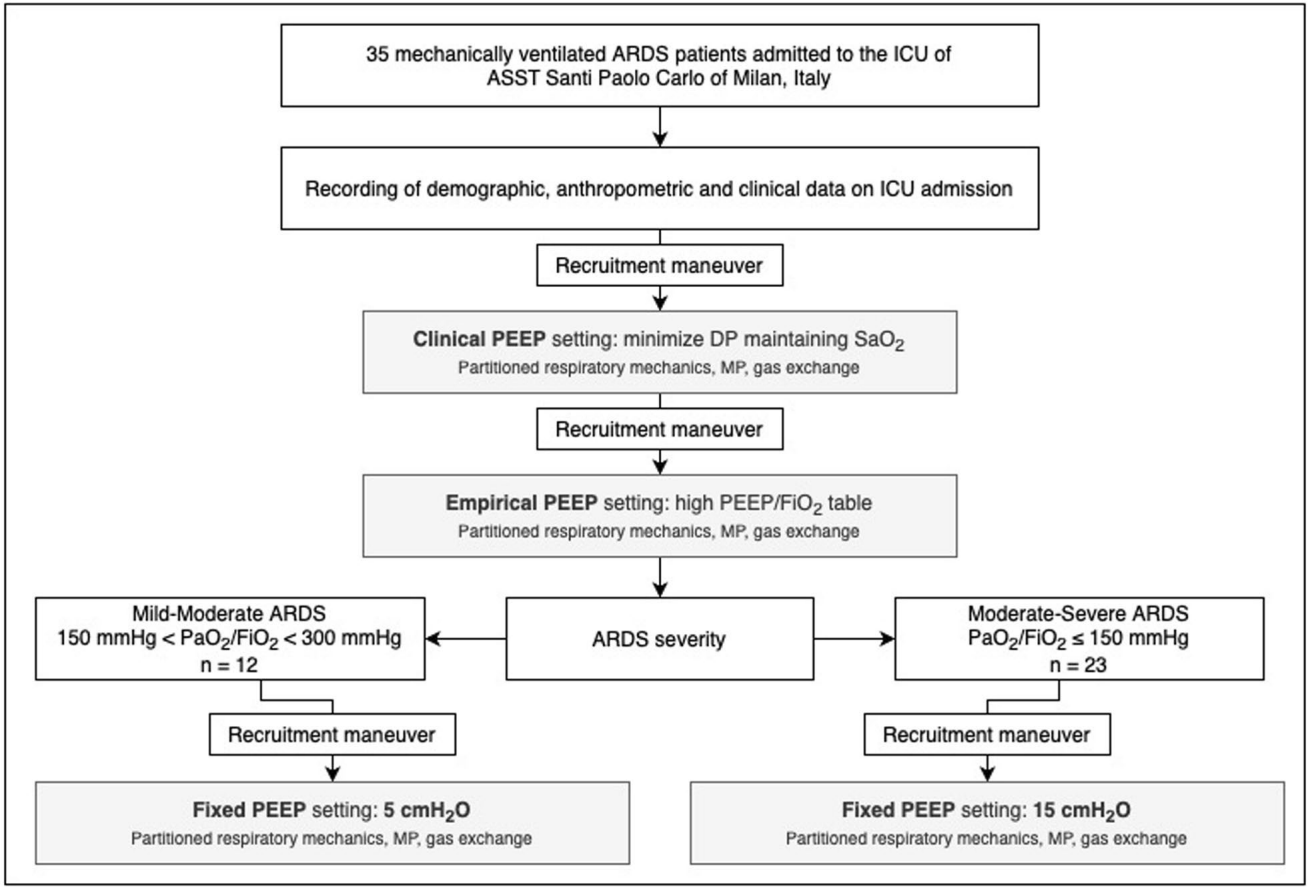
Study protocol flow-chart. ARDS: acute respiratory distress syndrome; DP: driving pressure; ICU: intensive care unit; PEEP: positive end-expiratory pressure

Thirty-five mechanically ventilated patients with ARDS according to the Berlin definition admitted to the general intensive care unit (ICU) of the ASST Santi Paolo Carlo, Milan, Italy were enrolled. Exclusion criteria were: presence of barotrauma, a history of severe chronic obstructive pulmonary disease and hemodynamic instability, as defined as a mean arterial pressure less than 70 mmHg despite fluid resuscitation and the use of vasopressors (i.e., norepinephrine). The study was approved by the local ethics committee (Comitato Etico Milano Area I; 2020/ST/095) and informed consent was obtained in accordance with Italian regulations. The study protocol is summarised in Fig. 1. The present study was not enlisted in any clinical trial registries.

### Study protocol

Mechanical ventilation was set in a volume-controlled mode with a tidal volume of 6–8 ml/Kg predicted body weight (PBW) during continuous deep sedation and paralysis. In each patient, the respiratory rate was set to achieve an arterial carbon dioxide partial pressure (PaCO_2_) of 40–50 mmHg with an inspiratory oxygen fraction (FiO_2_) to achieve a peripheral oxygen saturation (SpO_2_) of 92–96% at a PEEP of 5 cmH_2_O; during which time, the severity of ARDS was assessed [38]. Before each PEEP titration procedure, a recruitment maneuver (RM) was performed in pressure control mode with PEEP of 5 cmH_2_O, pressure above PEEP of 40 cmH_2_O, respiratory rate of 10 bpm, I/E ratio 1:1 and FiO_2_ of 0.7 for 2 min. Tidal volume, respiratory rate, fluid administration and catecholamine infusion were maintained unchanged throughout the study.

### Clinical PEEP setting according to driving pressure

In the study population, a decremental PEEP trial was performed starting from 15 cmH_2_O and decreasing by 1 cmH_2_O; the PEEP was selected as the level that provided the minimum driving pressure while maintaining SpO_2_ greater than 92% with FiO_2_ adjusted between 0.50 and 0.80.

### Empirical PEEP setting according to high PEEP/FiO_2_ table

In the study population, PEEP was titrated to maintain the lowest possible PEEP/FiO_2_ combination from an empirical table [13] to achieve an SpO_2_ between 88 and 93%.

### PEEP settings at 5 and 15 cmH_2_O

Two fixed PEEP levels were tested according to ARDS severity: PEEP of 5 cmH_2_O was set in mild-moderate ARDS patients (150 mmHg < PaO_2_/FiO_2_ < 300 mmHg) while a PEEP of 15 cmH_2_O was selected in moderate-severe ARDS patients (PaO_2_/FiO_2_ ≤ 150 mmHg).

Respiratory rate was not changed throughout the whole duration of the study.

### Lung CT scan

Before the beginning of the PEEP titration, two lung computed tomography scans were performed during an end-expiratory pressure hold at 5 cmH_2_O of PEEP and during an end-inspiratory pressure hold at 45 cmH_2_O of inspiratory airway pressure under static conditions [2]. Total lung weight, gas volume, and the proportions of the different compartments (not inflated, poorly inflated, well inflated and overinflated) were calculated using dedicated software (Maluna).

### Data collection

Twenty minutes after the application of the selected PEEP, partitioned respiratory mechanics, an arterial blood gas and hemodynamics were obtained. Partitioned respiratory mechanics were obtained using an esophageal catheter, positioned as already described elsewhere [37].

Additional material and methods are available in the Supplementary Digital Content.

### Statistical analysis

Continuous variables are expressed as mean ± SD or median [IQR], as appropriate (Shapiro–Wilk test), whereas categorical variables are expressed as numbers and percentages. Differences in terms of respiratory mechanics, gas exchange and hemodynamics within clinical and empirical PEEP levels in the whole population were assessed by a paired Student’s *t* test or a paired Wilcoxon-Mann–Whitney *U* test, as appropriate. Differences in terms of respiratory mechanics, gas exchange and hemodynamics within fixed, clinical and empirical PEEP levels in patients with mild-moderate or moderate-severe ARDS patients were assessed by a one-way analysis of variance (ANOVA) for repeated measures or a Friedman’s test, as appropriate. *Post-hoc* analyses were performed by pairwise comparisons with Bonferroni correction. Using end-inspiratory airway pressure as the primary statistical endpoint for sample size calculation, we estimated that a sample of 10 mild-moderate and 20 moderate-severe ARDS patients would give the study a power of 0.80 with a confidence level of 0.05 to detect a difference in end-inspiratory airway pressure of at least 20% among PEEP levels (clinical vs. empirical vs. fixed), estimated as the minimum clinically relevant difference assuming a mean end-inspiratory airway pressure of 15 cmH_2_O [2]. The behaviour of selected respiratory mechanics and gas exchange variables within fixed and clinical PEEP or fixed and empirical PEEP between mild-moderate and moderate-severe ARDS patients was assessed by a two-way repeated measures ANOVA, with PEEP titration method as *within* effect (*p*_*METHOD*_) and ARDS severity group as *between* effect (*p*_*SEVERITY*_); the interaction between the PEEP titration method and the ARDS severity group was reported as *p*_*INTER*_. The same procedure was used to investigate the influence of lung recruitability (*p*_RECRUIT_) by comparing patients with high vs. low lung recruitability, as defined as a value higher or lower than the median lung recruitability of the whole population. A *p* < 0.05 was considered as statistically significant.

## Results

Thirty-five ARDS patients were enrolled and their main clinical characteristics are shown in Table 1. Patients were assessed within 48 h after the ICU admission. At 5 cmH_2_O of PEEP, PaO_2_/FiO_2_ was 132 [82–158] mmHg and 12 (34%) and 23 (66%) patients had mild-moderate and moderate-severe form of ARDS respectively. At 5 cmH_2_O of PEEP, the mean total lung volume was 1198 ± 556 mL, with a total lung weight of 1472 ± 370 g; the amount of not aerated tissue was 620 [440–840] g with a median potential for lung recruitment of 20 [8–25] % (Table S1).

**Table 1:** Baseline characteristics of the study population

	*n* = 35
Demographics	
Age, years	66 ± 14
Male sex, *n* (%)	20 (57)
Weight, kg	81 ± 15
Height, m	1.68 ± 0.08
Body mass index, kg/m^2^	28.4 ± 4.5
SAPS II	33 ± 8
Diagnosis, *n* (%)	
Pancreatitis	2 (6)
Pneumonia	26 (74)
Sepsis	3 (9)
Septic shock	4 (11)
ARDS origin, *n* (%)	
Extrapulmonary	6 (17)
Pulmonary	29 (83)
PaO_2_/FiO_2_, mmHg	132 [82–158]
ARDS severity, *n* (%)	
Mild–moderate	12 (34)
Moderate–severe	23 (66)
Respiratory system elastance, cmH_2_O/l	24.6 ± 7.0
Outcome ICU, *n* (%)	
Alive	22 (63)
Dead	13 (37)
Outcome at 28 days, *n* (%)	
Alive	22 (63)
Dead	13 (37)
Outcome at hospital dimission, *n* (%)	
Alive	22 (63)
Dead	13 (37)
ICU length of stay, days	15 [12–19]
Ventilatory setting	
Tidal volume, ml	500 ± 45
Tidal volume per kg PBW, ml/kg	7.7 ± 0.8
Respiratory rate, bpm	16 ± 2
Minute ventilation, l/min	8.0 ± 1.5
PEEP, cmH_2_O	10 [8–10]
FiO_2_	0.60 ± 0.15

FiO_2_: inspired fraction of oxygen; ICU: intensive care unit; PaCO_2_: partial pressure of carbon dioxide; PaO_2_: partial pressure of oxygen; PBW: predicted body weight; PEEP: positive end-expiratory pressure; SAPS II: simplified acute physiology score

### Clinical versus empirical PEEP in the whole population

Table 2 shows data on respiratory mechanics, mechanical power and gas exchange at the two different PEEP levels in the whole population. Clinical and empirical PEEP were 10 [8–10] and 15 [10–18] cmH_2_O (*p* < 0.05). When comparing empirical to clinical PEEP, end-inspiratory airway pressure, lung and respiratory system elastances, mechanical power, lung stress and PaCO_2_ were significantly higher, but PaO_2_/FiO_2_ increased.

**Table 2:** Respiratory mechanics, gas exchange and hemodynamic variables according to clinical *versus* empirical PEEP titration strategy

*n* = 35	Clinical PEEP	Empirical PEEP	*p*
Respiratory mechanics			
PEEP, cmH_2_O	10 [8–10]	15 [10–18]	** < 0.001**
Mean airway pressure, cmH_2_O	14 [12–15]	20 [14–22]	** < 0.001**
Peak pressure, cmH_2_O	30 ± 7	36 ± 8	** < 0.001**
End-inspiratory airway pressure, cmH_2_O	21 ± 4	27 ± 7	** < 0.001**
Driving pressure, cmH_2_O	12 ± 3	13 ± 4	**0.037**
Respiratory system elastance, cmH_2_OLl	24.6 ± 7.0	26.8 ± 8.2	**0.013**
Chest wall elastance, cmH_2_O/L	5.7 [3.9–7.6]	5.2 [3.5–7.1]	**0.019**
Lung elastance, cmH_2_O/L	18.6 ± 6.7	21.1 ± 8.1	** < 0.001**
End-expiratory transpulmonary pressure, cmH_2_O	− 3.8 ± 4.9	− 0.5 ± 5.1	** < 0.001**
End-inspiratory transpulmonary pressure, cmH_2_O	5.3 ± 4.0	10.0 ± 6.0	** < 0.001**
Lung stress, cmH_2_O	15.9 ± 4.1	21.3 ± 6.6	** < 0.001**
Transpulmonary driving pressure, cmH_2_O	9.1 ± 3.1	10.5 ± 3.8	** < 0.001**
Mechanical power, J/min	16.9 [14.3–23.0]	23.6 [19.3–28.1]	** < 0.001**
Gas exchange			
FiO_2_	0.60 [0.50–0.75]	0.40 [0.40–0.55]	** < 0.001**
PaO_2_/FiO_2_, mmHg	137 [98–163]	181 [139–205]	** < 0.001**
Arterial pH	7.37 ± 0.06	7.36 ± 0.06	0.119
PaO_2_, mmHg	74 [70–84]	74 [69–94]	0.416
PaCO_2_, mmHg	44 [42–48]	47 [44–53]	**0.013**
Ventilatory ratio	1.46 [1.22–1.68]	1.48 [1.34–1.82]	**0.002**
SpO_2_, %	94 [93–96]	95 [93–96]	0.805
Hemodynamics			
Lactates, mmol/L	1.2 [0.9–1.4]	1.2 [1.0–1.3]	0.562
Systolic arterial pressure, mmHg	131 ± 17	126 ± 19	0.099
Diastolic arterial pressure, mmHg	63 ± 12	63 ± 10	0.473
Mean arterial pressure, mmHg	85 ± 14	83 ± 11	0.330
Heart rate, bpm	82 ± 17	82 ± 19	0.759
Central venous pressure, mmHg	11 ± 4	13 ± 4	** < 0.001**

FiO_2_: inspired fraction of oxygen; PaCO_2_: partial pressure of carbon dioxide; PaO_2_: partial pressure of oxygen; PBW: predicted body weight; PEEP: positive end-expiratory pressure; SpO_2_: peripheral oxygen saturation. Bold: *p* < 0.050

### Fixed versus clinical versus empirical PEEP within mild-moderate and moderate-severe ARDS

**Fig. 2:**
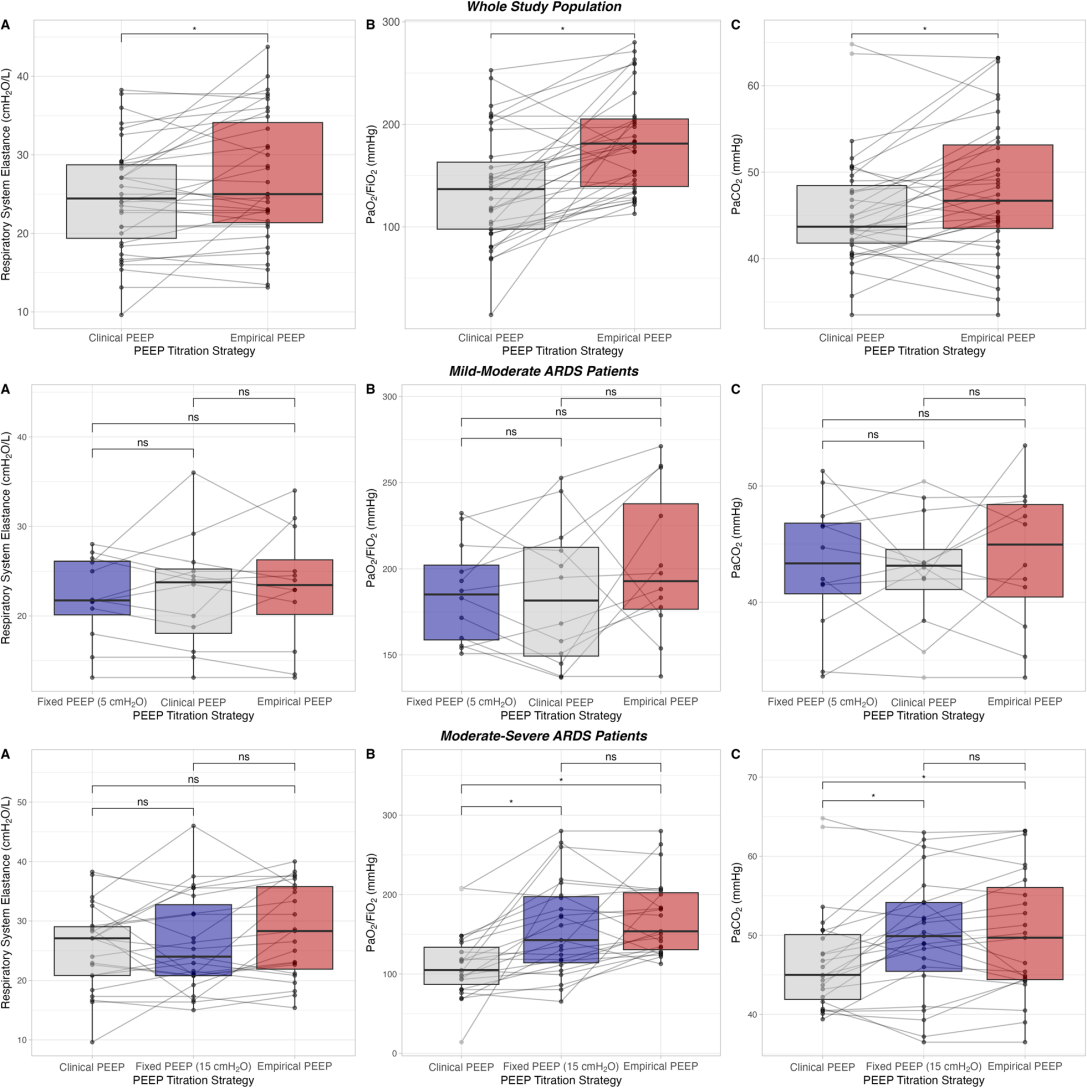
Respiratory system elastance **A**, PaO_2_/FiO_2_ ratio **B** and arterial carbon dioxide partial pressure (PaCO_2_, **C** according to PEEP titration strategies in the whole population (upper panel, clinical *versus* empirical PEEP), in mild-moderate ARDS patients (middle panel, fixed 5 cmH_2_O *versus* clinical versus empirical PEEP) and in moderate severe ARDS patients (lower panel, fixed 5 cmH_2_O *versus* clinical *versus* empirical PEEP). *: *p* < 0.050; ns: non statistically significant

In mild-moderate ARDS patients, all PEEP levels were significantly different when comparing clinical PEEP (8 [8–10] cmH_2_O) with a fixed PEEP of 5 cmH_2_O and empirical PEEP (10 [8–12]) (*p* < 0.05). End-inspiratory airway pressure and lung stress were significantly higher with clinical and empirical PEEP, whereas gas exchange, lung and chest elastance were not different (Table 3, Fig. 2).

**Table 3:** Respiratory mechanics, gas exchange and hemodynamic variables according to clinical, empirical or fixed (5 cmH_2_O in mild-moderate or 15 cmH_2_O in moderate-severe ARDS patients) PEEP titration strategies in mild-moderate ARDS patients (PaO_2_/FiO_2_ at 5 cmH_2_O of PEEP between 150 and 300 mmHg)

*n* = 12	Fixed PEEP	Clinical PEEP	Empirical PEEP	*p*
Mild-Moderate ARDS (150 mmHg < PaO_2_/FiO_2_ ≤ 300 mmHg)				
Respiratory mechanics				
PEEP, cmH_2_O	5	8 [8–10]*	10 [8–12]*°	–
Mean airway pressure, cmH_2_O	10 ± 2	14 ± 4*	15 ± 4*	**0.003**
Peak pressure, cmH_2_O	28 ± 8	31 ± 9	34 ± 10*	**0.003**
End-inspiratory airway pressure, cmH_2_O	16 ± 2	20 ± 4*	23 ± 7*	**0.007**
Driving pressure, cmH_2_O	11 ± 2	12 ± 3	12 ± 4	0.442
Respiratory system elastance, cmH_2_O/L	22.1 ± 4.7	22.6 ± 6.4	24.0 ± 8.4	0.464
Chest wall elastance, cmH_2_O/L	5.1 ± 2.3	4.9 ± 2.0	4.5 ± 1.8	0.124
Lung elastance, cmH_2_O/L	17.0 ± 4.7	17.8 ± 6.0	19.5 ± 7.9	0.349
End-expiratory transpulmonary pressure, cmH_2_O	-5.5 ± 6.0	-3.1 ± 5.1*	-1.6 ± 5.9*	**0.012**
End-inspiratory transpulmonary pressure, cmH_2_O	3.3 ± 4.3	6.0 ± 3.9*	8.5 ± 6.5*	**0.031**
Lung stress, cmH_2_O	12.6 ± 2.7	15.6 ± 4.0*	18.8 ± 6.8*	**0.013**
Transpulmonary driving pressure, cmH_2_O	8.8 ± 2.3	9.1 ± 3.0	10.1 ± 3.9	0.319
Mechanical Power, J/min	18.6 ± 8.4	20.3 ± 10.2	23.6 ± 10.0*	**0.003**
Gas exchange				
FiO_2_	0.45 [0.40–0.50]	0.50 [0.40–0.60]	0.40 [0.40–0.40]	0.065
PaO_2_/FiO_2_, mmHg	186 ± 29	185 ± 41	203 ± 43	0.241
Arterial pH	7.38 ± 0.05	7.39 ± 0.05	7.38 ± 0.06	0.626
PaO_2_, mmHg	83 ± 13	88 ± 17	81 ± 20	0.316
PaCO_2_, mmHg	43 ± 6	43 ± 5	44 ± 6	0.458
Ventilatory ratio	1.39 ± 0.18	1.36 ± 0.24	1.42 ± 0.23	0.826
SpO_2_, %	95 ± 2	96 ± 1	94 ± 3	0.074
Hemodynamics				
Lactates, mmol/l	1.2 ± 0.2	1.2 ± 0.3	1.2 ± 0.2	0.616
Systolic arterial pressure, mmHg	129 ± 21	133 ± 13	129 ± 20	0.313
Diastolic arterial pressure, mmHg	60 ± 12	62 ± 10	60 ± 10	0.112
Mean arterial pressure, mmHg	82 ± 16	85 ± 12	81 ± 12	0.330
Heart rate, bpm	72 ± 13	74 ± 14	74 ± 15	0.759
Central venous pressure, mmHg	10 ± 3	11 ± 3*	11 ± 3*	**0.003**

FiO_2_: inspired fraction of oxygen; PaCO_2_: partial pressure of carbon dioxide; PaO_2_: partial pressure of oxygen; PBW: predicted body weight; PEEP: positive end-expiratory pressure; SpO_2_: peripheral oxygen saturation. *: *p* < 0.050 vs. Fixed PEEP; °: *p* < 0.050 vs. Clinical PEEP. Bold: *p* < 0.050

In moderate-severe ARDS patients, when comparing clinical PEEP (10 [8–10] cmH_2_O) with a fixed PEEP of 15 cmH_2_O and empirical PEEP (17 [14–18] cmH_2_O), empirical and fixed PEEP were similar, but both higher than clinical PEEP. End-inspiratory airway pressure, lung and chest wall elastances and mechanical power were similar between fixed PEEP and empirical PEEP, but worse with respect to clinical PEEP. Oxygenation was significantly higher with fixed and empirical PEEP as compared to clinical PEEP, whereas PaCO_2_ and ventilatory ratio were lower with clinical PEEP. Hemodynamics were not affected by the changes of PEEP (Table 4, Fig. 2).

**Table 4:** Respiratory mechanics, gas exchange and hemodynamic variables according to clinical, empirical or fixed (5 cmH_2_O in mild-moderate or 15 cmH_2_O in moderate-severe ARDS patients) PEEP titration strategies in moderate-severe ARDS patients (PaO_2_/FiO_2_ at 5 cmH_2_O of PEEP < 150 mmHg)

*n* = 23	Fixed PEEP	Clinical PEEP	Empirical PEEP	*p*
Moderate-Severe ARDS (PaO_2_/FiO_2_ ≤ 150 mmHg)				
Respiratory mechanics				
PEEP, cmH_2_O	15	10 [8–10]*	17 [14–18]	–
Mean airway pressure, cmH_2_O	20 [19–21]	14 [13–15]*	21 [18–22]	** < 0.001**
Peak pressure, cmH_2_O	37 ± 6	29 ± 6*	37 ± 60°	** < 0.001**
End-inspiratory airway pressure, cmH_2_O	28 ± 4	22 ± 4*	29 ± 5°	** < 0.001**
Driving pressure, cmH_2_O	13 ± 4	12 ± 3	14 ± 3°	**0.019**
Respiratory system elastance, cmH_2_O/L	26.2 ± 8.3	25.6 ± 7.2	28.3 ± 7.8	0.053
Chest wall elastance, cmH_2_O/L	6.4 ± 3.4	6.5 ± 3.2	6.3 ± 3.5	0.748
Lung elastance, cmH_2_O/L	19.8 ± 8.4	19.1 ± 7.1	22.0 ± 8.2°	**0.033**
End-expiratory transpulmonary pressure, cmH_2_O	− 0.1 ± 5.1	− 4.2 ± 5.0*	0.1 ± 4.6°	** < 0.001**
End-inspiratory transpulmonary pressure, cmH_2_O	9.6 ± 6.2	4.9 ± 5.5*	10.8 ± 5.7°	** < 0.001**
Lung stress, cmH_2_O	20.8 ± 5.8	16.1 ± 4.3*	22.6 ± 6.3*°	** < 0.001**
Transpulmonary driving pressure, cmH_2_O	9.8 ± 4.1	9.2 ± 3.2	10.7 ± 3.9°	**0.014**
Mechanical power, J/min	24.7 ± 6.5	18.0 ± 5.6*	24.5 ± 6.8°	** < 0.001**
Gas exchange				
FiO_2_	0.65 [0.55–0.80]	0.70 [0.60–0.80]	0.50 [0.40–0.60]*°	** < 0.001**
PaO_2_/FiO_2_, mmHg	162 ± 69	112 ± 43*	171 ± 48°	** < 0.001**
Arterial pH	7.35 ± 0.05	7.36 ± 0.06	7.35 ± 0.06	0.058
PaO_2_, mmHg	93 [74–111]	72 [62–76]*	74 [70–94]*°	**0.011**
PaCO_2_, mmHg	50 [45–54]	45 [42–50]*	50 [44–56]°	**0.039**
Ventilatory ratio	1.67 ± 0.35	1.52 ± 0.29*	1.69 ± 0.42°	**0.001**
SpO_2_, %	96 ± 2	93 ± 3*	94 ± 3*	** < 0.001**
Hemodynamics				
Lactates, mmol/l	1.2 ± 0.3	1.1 ± 0.3	1.1 ± 0.3	0.099
Systolic arterial pressure, mmHg	125 ± 21	130 ± 19	124 ± 18	0.357
Diastolic arterial pressure, mmHg	64 ± 13	64 ± 13	64 ± 10	0.952
Mean arterial pressure, mmHg	85 ± 13	85 ± 15	84 ± 11	0.961
Heart rate, bpm	89 ± 20	87 ± 17	86 ± 19	0.410
Central venous pressure, mmHg	13 ± 4	11 ± 4*	13 ± 4°	**0.004**

FiO_2_: inspired fraction of oxygen; PaCO_2_: partial pressure of carbon dioxide; PaO_2_: partial pressure of oxygen; PBW: predicted body weight; PEEP: positive end-expiratory pressure; SpO_2_: peripheral oxygen saturation. *: *p* < 0.050 vs. Fixed PEEP; °: *p* < 0.050 vs. Clinical PEEP. Bold:* p* < 0.050

### Response to fixed versus empirical PEEP between severity classes

The change from fixed to empirical PEEP increased end-inspiratory airway pressure and mechanical power in mild-moderate ARDS patients, whereas it did not affect these variables in moderate-severe ARDS patients. However, the change in PEEP from fixed to empirical resulted in a similar change in term of elastances, gas exchange and ventilatory ratio both in mild-moderate and in moderate-severe ARDS patients, although the increase in lung stress was lower in mild-moderate compared to moderate-severe ARDS patients (*p*_*INTER*_ = 0.023) (Table S2).

### Response to fixed versus clinical PEEP between severity classes

The change in PEEP from fixed to clinical worsened end-inspiratory airway pressure and stress in mild-moderate ARDS patients whereas it decreased end-inspiratory airway pressure and stress in moderate-severe ARDS patients, with similar responses in elastances and PaCO_2_ between groups. While the change in PEEP from fixed to clinical did not affect oxygenation and mechanical power in mild-moderate ARDS patients, it decreased oxygenation and mechanical power in moderate-severe ARDS patients (Table S3).

### Response to clinical versus empirical PEEP according to lung recruitability

Patients with low and high lung recruitability exhibited 4 ± 1 and 27 ± 7% of lung recruitability, respectively (Table S4).

The response to the change in PEEP from clinical to empirical was similar in patients with low and high potential for lung recruitment in terms of respiratory mechanics and gas exchange (Table S4).

## Discussion

The main findings of this study, evaluating different PEEP titration strategies in ARDS patients, during controlled mechanical ventilation, were: (1) regardless of the severity, the use of empirical PEEP worsened respiratory mechanics and PaCO_2_ but improved oxygenation compared with clinical PEEP, (2) in mild-moderate ARDS patients, the use of empirical or clinical PEEP resulted in higher PEEP levels, worse respiratory mechanics and similar gas exchange compared with a low fixed PEEP level (3) in moderate-severe ARDS patients, the use of empirical and high fixed PEEP levels resulted in similar PEEP levels, respiratory mechanics and gas exchange whereas clinical PEEP resulted in better respiratory mechanics and CO_2_ clearance but with worse oxygenation.

The present study evaluated three different methods of PEEP selection, without tidal volume modification in mechanically ventilated ARDS patients to assess the effects of PEEP changes both on the amount of the static component of the mechanical power and indirectly on the driving pressure and the respiratory mechanics. Three methods of PEEP selection were compared: a clinical PEEP based on driving pressure and an empirical PEEP according to a high PEEP/FiO_2_ table in the whole population, and two fixed levels of PEEP according to severity (PEEP 5 cmH_2_O when PaO_2_/FiO_2_ 150–300 mmHg and PEEP 15 cmH_2_O when PaO_2_/FiO_2_ ≤ 150 mmHg) [13].

Fifteen cmH_2_O was chosen as the high PEEP level because 12 cmH_2_O of PEEP has been proposed as a threshold for defining high versus low PEEP and also because PEEP levels higher than 15 cmH_2_O have been associated with increased in the mortality [21, 39]. The empirical method is one of the most commonly investigated methods in RCTs and has been shown to individualise PEEP according to lung recruitability compared to other PEEP selection methods [4, 9]. In the present study the clinical PEEP was 10 [8–10] cmH_2_O, similar to the values found in the Lung SAFE study, which included 2377 patients invasively mechanically ventilated ARDS patients where the target PEEP was 8.4 (8.3–8.6) cmH_2_O [40]. In the whole population, empirical PEEP titration resulted in a higher PEEP level as compared to the clinical driving pressure-based method, resulting in an increased end-inspiratory airway pressure in empirical PEEP (21 ± 4 vs. 27 ± 7 cmH_2_O), without a clinical benefit in terms of driving pressure. Empirical PEEP titration was also associated with the worsening of other VILI indices, such as lung and respiratory system elastances, lung stress and mechanical power, with a consequent worsening of CO_2_ clearance, despite an improvement in oxygenation.

In mild-moderate ARDS patients, clinical and empirical PEEP titration increased end-inspiratory airway pressure as compared with a low fixed PEEP level at similar driving pressures. In contrast, in patients with moderate-severe ARDS, empirical and a high fixed PEEP titration resulted in a higher end-inspiratory airway pressure with a concomitant increase in driving pressure, suggesting that PEEP titration in more severe forms of ARDS should include assessment of respiratory mechanics.

Increasing PEEP while maintaining a constant tidal volume may increase both the basal strain and the end-inspiratory stress depending on lung recruitability and respiratory mechanics, thus increasing the risk of VILI [41, 42].

The increase in the mechanical power was mainly due to an increase in the static component (*i.e.,* PEEP).

In an ultra-protective ventilatory strategy in ECMO patients, Boesing et al., evaluating different PEEP strategies found that PEEP selected according to the ELSO recommendations was significantly lower compared to a decremental PEEP trial, with also lower values of mechanical power and lung stress [35]. PEEP selection according to a decremental PEEP trial compared to a PEEP/FiO_2_ table significantly reduced the mechanical power with a significant change (i.e., reduction) in PEEP [14].

A previous experimental study found that mechanical power did not change with PEEP levels between 0 and 7 cmH_2_O because the increase in PEEP was offset by the concomitant decrease in driving pressure [34]. In contrast, when PEEP was between 11 and 18 cmH_2_O mechanical power increased due to the PEEP component while the driving pressure did not change.

In our small population, regardless of ARDS severity, PEEP titration based on driving pressure resulted in lower PEEP levels with better mechanics and CO_2_ clearance compared with empirical PEEP. When we stratified according to ARDS severity, a low fixed level of PEEP appeared to be appropriate in mild-moderate patients, as titration using empirical or clinical PEEP provided no benefit, both resulting in higher PEEP levels with worse respiratory mechanics and similar gas exchange. Conversely, in moderate-severe ARDS patients, titration using empirical and high fixed PEEP levels did not provide any benefit, as these led to similar PEEP levels, respiratory mechanics and gas exchange, whereas clinical PEEP led to lower PEEP levels, resulting in safer values in terms of respiratory mechanics with better CO_2_ clearance but worse oxygenation.

In contrast, in a population characterised by low levels of oxygenation, such as ARDS COVID-19 patients, the empirical PEEP set according to the PEEP/FiO_2_ table was significantly higher than a fixed 15 cmH_2_O when comparing different PEEP criteria [37].

Finally, we did not find any difference in the main hemodynamic parameters within the PEEP titration strategies, probably due to the patients’ fluid status and catecholamine optimisation before the start of the study.

The limitations of our study are: firstly, a small population; secondly, the order of application of the different PEEP titration strategies was not randomised, although the 20-min interval between steps and the use of recruitment maneuvers reduced the possibility of a carry-over effect [43]; thirdly, the possible influence of confounding factors, such as the presence of airway opening pressures, which was not studied but which could have influenced respiratory mechanics at different PEEP levels; however, even if present, its effect did not seem to be relevant due to better respiratory mechanics at low PEEP levels.

## Conclusions

This study showed that clinical PEEP titration provided better respiratory mechanics and better CO_2_ clearance than a high PEEP/FiO_2_ table, regardless of ARDS severity. In mild-moderate ARDS patients, a low fixed PEEP level (5 cmH_2_O) provided better mechanics without any detrimental effects on gas exchange compared to clinical or empirical PEEP. In moderate-severe ARDS patients, there appears to be no benefit from empirical PEEP or a high fixed PEEP level (15 cmH_2_O) compared with more moderate PEEP levels set according to clinician judgement. Our study suggests that PEEP titration in more severe ARDS patients should take into account respiratory mechanics. 

## Supplementary Information

Supplementary Material 1.

## Data Availability

No datasets were generated or analysed during the current study.
