# Supplementary material for: Driving pressure vs. oxygenation-based PEEP titration strategies in ARDS patients: a physiological study
Source: Crit Care. 2025 Jul 8;29:289. doi: 10.1186/s13054-025-05459-8 (PMC12239453; doi:10.1186/s13054-025-05459-8)
Supplement: Supplementary file 1 — Supplementary Material 1. [file 13054_2025_5459_MOESM1_ESM.docx]

**EFFECTS OF PEEP TITRATION STRATEGIES IN ARDS PATIENTS**

*Supplementary Material*

^1^ Department of Health Sciences, University of Milan, Italy

^2^ Department of Anesthesia and Intensive Care, ASST Santi Paolo e Carlo, San Paolo University Hospital Milan, Italy

^3^ Coordinated Research Center on Respiratory Failure, University of Milan, Italy

*Additional Methods*

To measure the esophageal pressure, a radio-opaque balloon catheter (SmartCath Bicore, USA) was placed in the lowest part of the esophagus and connected to a pressure transducer. The esophageal catheter was inflated with 1.5 mL of air and inserted trans-orally to reach the stomach at a depth between 50 - 55 cm from the mouth^1^. The intragastric position was confirmed by a rise in intra-abdominal pressure after external manual epigastric compression. Then, it was withdrawn into the esophagus (*i.e.,* confirmed by the presence of cardiac artefacts in the pressure tracing and by the difference in the absolute pressure compared the intragastric pressure) at a distance between 35 and 40 cm from the mouth.

For the CT quantitative analysis, the lung profiles of each CT scan slice were manually contoured, excluding hilar structures. Then, quantitative analysis was performed using a dedicated software, *i.e.,* Maluna^2^, which computed the lung weight, lung gas volume, amount of over-inflated tissue (voxel density − 1000 to − 900 Hounsfield Units, HU), well-aerated tissue (− 899 to − 500 HU), poorly aerated tissue (− 499 to − 100 HU) and non-aerated tissue (− 100 to + 100 HU). Lung recruitability and overinflation were, respectively, computed as the ratio between the difference in non-aerated tissue at 5 cmH_2_O of PEEP and at 45 cmH_2_O of PEEP and the total lung tissue at 5 cmH_2_O of PEEP and as the ratio between the difference in well inflated tissue at 45 cmH_2_O of PEEP and at 5 cmH_2_O of PEEP and the total lung tissue at 5 cmH_2_O of PEEP.^3^

*Measurements*

*Respiratory Mechanics*

Respiratory system mechanics was assessed by performing end-inspiratory and end-expiratory airway occlusion maneuvers of 5 seconds each, to measure end-inspiratory airway pressure and total PEEP, respectively.

The following variables were consequently calculated:

$$Driving Pressure \left( cmH_{2}O \right)=End inspiratory airway pressure-total PEEP$$

$$Respiratory System Elastance \left( E_{RS}, \frac{cmH_{2}O}{L} \right)=\frac{Driving Pressure}{Tidal Volume (L)}$$

Partitioned respiratory mechanics was obtained by measuring end-inspiratory and end-expiratory esophageal pressures, as already described.^4^ The respiratory system includes the lung and the chest wall, in series; therefore, the overall mechanical behavior mathematically depends on the mechanical characteristics of the lung and the chest wall and their interactions. The term partitioned respiratory mechanics refers to the dividing the respiratory system in its components, i.e., the lung and the chest wall.^4^

The following variables were consequently calculated:

$$\Delta Esophageal Pressure \left( \Delta Pes, cmH_{2}O \right)=End inspiratory Pes-End expiratory Pes$$

$$Chest Wall Elastance \left( E_{CW}, \frac{cmH_{2}O}{L} \right)=\frac{\Delta Pes}{Tidal Volume (L)}$$

Lung elastance and lung stress were consequently obtained:

$$Lung Elastance \left( E_{L}, \frac{cmH_{2}O}{L} \right)=E_{RS}-E_{CW}$$

$$Lung Stress \left( cmH_{2}O \right)=End inspiratory airway pressure\times\frac{E_{L}}{E_{RS}}$$

Transpulmonary driving pressure were obtained according to Chiumello *et al:* ^5^

$$Transpulmonary Driving Pressure \left( cmH_{2}O \right)=Driving Pressure\times\frac{E_{L}}{E_{RS}}$$

The mechanical power was calculated based on a mathematical simplification of the original mechanical power for volume control ventilated patients: ^6,7^

$$MP=0.098\times V_{T}\times RR\times(Ppeak- \frac{Driving Pressure}{2})$$

where V_T_ is tidal volume, RR is respiratory rate, Ppeak is peak airway pressure.

*Gas Exchange*

Ventilatory ratio was calculated according to Sinha *et al:^8^*

$$Ventilatory Ratio=\frac{V_{E measured}\times{PaCO}_{2 measured}}{V_{E predicted}\times{PaCO}_{2 ideal}}$$

Where V_E measured_ is measured minute ventilation (L/min); PaCO_2 measured_ is measured arterial carbon dioxide partial pressure; V_E predicted_ is predicted minute ventilation (L/min), calculated as 0.1L/min*PBW and PaCO_2 ideal_ is ideal arterial carbon dioxide partial pressure, assumed to be 40 mmHg.

**Table S1.** Lung CT scan data at 5 and 45 cmH_2_O of airway pressure in the whole study population.

|  | **PEEP**  **5 cmH_2_O** | **PEEP**  **45 cmH_2_O** | *p* |
| --- | --- | --- | --- |
| Total lung volume, ml | 2655 ± 782 | 4081 ± 1111 | **<0.001** |
| Total tissue volume, ml | 1457 ± 368 | 1488 ± 365 | 0.398 |
| Total tissue mass, g | 1472 ± 370 | 1497 ± 367 | 0.490 |
| Total gas volume, ml | 1198 ± 556 | 2593 ± 936 | **<0.001** |
| Overinflated volume, ml | 13.7 [1.89 - 29.2] | 367 [47.7 - 609] | **<0.001** |
| Overinflated mass, g | 1.1 [0.2 - 2.4] | 29.1 [3.9 - 42.6] | **<0.001** |
| Overinflated fraction, % | 0.17 ± 0.3 | 2.1 ± 2.1 | **<0.001** |
| Normally aerated volume, ml | 1351 ± 684 | 2603 ± 841 | **<0.001** |
| Normally aerated mass, g | 375 ± 166 | 622 ± 204 | **<0.001** |
| Normally aerated fraction, % | 26.2 ± 11.1 | 42.3 ± 12.3 | **<0.001** |
| Poorly aerated volume, ml | 588 ± 359 | 606 ± 315 | 0.051 |
| Poorly aerated mass, g | 412 ± 268 | 408 ± 215 | 0.106 |
| Poorly aerated fraction, % | 27.2 ± 13.2 | 26.6 (9.6) | 0.199 |
| Not aerated volume, ml | 616 [438 – 848] | 379 [244 - 534] | **<0.001** |
| Not aerated mass, g | 682 ± 305 | 437 ± 293 | **<0.001** |
| Not aerated fraction, % | 46.5 ± 15.9 | 29.0 ± 17.2 | **<0.001** |
| Potential for lung recruitment, % | 20 [8 - 25] | | **-** |

**Table S2**. Behavior of ARDS patients during PEEP titration according to fixed or empirical strategy and ARDS severity. FiO_2_: inspired fraction of oxygen; PaCO_2_: partial pressure of carbon dioxide; PaO_2_: partial pressure of oxygen; PEEP: positive end-expiratory pressure.

*: *p* < 0.050 *vs* Mild-Moderate ARDS; °: *p* < 0.050 *vs* Fixed PEEP.

|  | Mild-Moderate ARDS  n = 12 | Moderate-Severe  ARDS  n = 23 | *p_SEVERITY_* | *p_METHOD_* | *p_INTER_* |
| --- | --- | --- | --- | --- | --- |
| PEEP, *cmH_2_O*  Fixed PEEP  Empirical PEEP | 5  10 [8 – 12] | 15  17 [14 – 18]* | *-* | *-* | *-* |
| Peak pressure, *cmH_2_O*  Fixed PEEP  Empirical PEEP | 28 ± 8  34 ± 10° | 37 ± 6*  37 ± 6 | ***0.015*** | ***0.002*** | ***0.004*** |
| End-inspiratory airway pressure, *cmH_2_O*  Fixed PEEP  Empirical PEEP | 16 ± 2  23 ± 7° | 28 ± 4*  29 ± 5* | ***<0.001*** | ***<0.001*** | ***0.009*** |
| Driving pressure, *cmH_2_O*  Fixed PEEP  Empirical PEEP | 11 ± 2  12 ± 4 | 13 ± 4  14 ± 3 | *0.231* | ***0.046*** | *0.949* |
| E_RS_, *cmH_2_O/L*  Fixed PEEP  Empirical PEEP | 22 ± 5  24 ± 8 | 26 ± 8  28 ± 8 | *0.106* | *0.051* | *0.933* |
| E_L_, *cmH_2_O/L*  Fixed PEEP  Empirical PEEP | 17 ± 5  20 ± 8 | 20 ± 8  22 ± 8 | *0.308* | ***0.032*** | *0.873* |
| End-expiratory transpulmonary pressure, *cmH_2_O/L*  Fixed PEEP  Empirical PEEP | -5.5 ± 5.9  -1.6 ± 5.9° | -0.1 ± 5.1*  0.1 ± 4.6 | ***0.053*** | ***0.002*** | ***0.005*** |
| End-inspiratory transpulmonary pressure, *cmH_2_O/L*  Fixed PEEP  Empirical PEEP | 3.3 ± 4.3  8.5 ± 6.5° | 9.6 ± 6.2*  10.8 ± 5.7 | ***0.028*** | ***<0.001*** | ***0.025*** |
| Lung stress, *cmH_2_O*  Fixed PEEP  Empirical PEEP | 13 ± 3  19 ± 7° | 21 ± 6*  23 ± 6° | ***0.003*** | ***0.001*** | ***0.023*** |
| Mechanical Power, *J/min*  Fixed PEEP  Empirical PEEP | 18.6 ± 8.4  23.6 ± 10.0° | 24.7 ± 6.5*  24.5 ± 6.8 | *0.187* | ***0.002*** | ***0.001*** |
| Arterial PO_2_, *mmHg*  Fixed PEEP  Empirical PEEP | 81 ± 12  76 ± 13 | 92 ± 25  79 ± 17 | *0.258* | ***0.031*** | *0.309* |
| Arterial PCO_2_, *mmHg*  Fixed PEEP  Empirical PEEP | 43 ± 6  44 ± 6 | 50 ± 8  50 ± 8 | ***0.016*** | *0.336* | *0.617* |
| Ventilatory Ratio  Fixed PEEP  Empirical PEEP | 1.4 ± 0.2  1.4 ± 0.2 | 1.7 ± 0.3  1.7 ± 0.4 | ***0.022*** | *0.442* | *0.959* |

**Table S3**. Behavior of ARDS patients during PEEP titration according to fixed or empirical strategy and ARDS severity. FiO_2_: inspired fraction of oxygen; PaCO_2_: partial pressure of carbon dioxide; PaO_2_: partial pressure of oxygen; PEEP: positive end-expiratory pressure.

*: *p* < 0.050 *vs* Mild-Moderate ARDS; °: *p* < 0.050 *vs* Fixed PEEP.

|  | Mild-Moderate  ARDS  n = 12 | Moderate-Severe  ARDS  n = 23 | *p_SEVERITY_* | *p_METHOD_* | *p_INTER_* |
| --- | --- | --- | --- | --- | --- |
| PEEP, *cmH_2_O*  Fixed PEEP  Clinical PEEP | 5  8 [8 – 10] | 15  10 [8 – 10] | *-* | *-* | *-* |
| Peak pressure, *cmH_2_O*  Fixed PEEP  Clinical PEEP | 28 ± 8  31 ± 9 | 37 ± 6*  29 ± 6° | *0.106* | ***<0.001*** | ***<0.001*** |
| End-inspiratory airway pressure, *cmH_2_O*  Fixed PEEP  Clinical PEEP | 16 ± 2  20 ± 4° | 28 ± 4*  22 ± 4° | ***<0.001*** | ***0.017*** | ***<0.001*** |
| Driving pressure, *cmH_2_O*  Fixed PEEP  Clinical PEEP | 11 ± 2  11 ± 6 | 13 ± 4  12 ± 3 | *0.309* | *0.661* | *0.436* |
| E_RS_, *cmH_2_O/L*  Fixed PEEP  Clinical PEEP | 22 ± 5  23 ± 6 | 26 ± 8  26 ± 7 | *0.136* | *0.973* | *0.570* |
| E_L_, *cmH_2_O/L*  Fixed PEEP  Clinical PEEP | 17 ± 5  18 ± 6 | 20 ± 8  19 ± 7 | *0.383* | *0.963* | *0.454* |
| End-expiratory transpulmonary pressure, *cmH_2_O/L*  Fixed PEEP  Clinical PEEP | -5.5 ± 6.0  -3.1 ± 5.1° | -0.1 ± 5.1*  -4.2 ± 5.0° | *0.252* | ***0.036*** | ***<0.001*** |
| End-inspiratory transpulmonary pressure, *cmH_2_O/L*  Fixed PEEP  Clinical PEEP | 3.3 ± 4.3  6.0 ± 3.9° | 9.6 ± 6.2*  4.9 ± 5.5° | *0.161* | *0.061* | ***<0.001*** |
| Lung stress, *cmH_2_O*  Fixed PEEP  Clinical PEEP | 13 ± 3  16 ± 4° | 21 ± 6*  16 ± 4° | ***0.007*** | *0.196* | ***<0.001*** |
| Mechanical Power, *J/min*  Fixed PEEP  Clinical PEEP | 18.4 ± 8.6  20.3 ± 10.2 | 24.3 ± 6.4*  18.0 ± 5.6° | *0.458* | ***0.003*** | ***<0.001*** |
| Arterial PO_2_, *mmHg*  Fixed PEEP  Clinical PEEP | 83 ± 13  88 ± 16 | 91 ± 25*  71 ± 10° | *0.400* | ***0.048*** | ***0.002*** |
| Arterial PCO_2_, *mmHg*  Fixed PEEP  Clinical PEEP | 43 ± 6  43 ± 5 | 50 ± 8  47 ± 7 | ***0.021*** | *0.051* | *0.157* |
| Ventilatory Ratio  Fixed PEEP  Clinical PEEP | 1.4 ± 0.2  1.4 ± 0.2 | 1.7 ± 0.3*  1.5 ± 0.3° | ***0.037*** | ***0.002*** | ***0.029*** |

**Table S4**. Behavior of ARDS patients during PEEP titration according to fixed or empirical strategy and ARDS severity. FiO_2_: inspired fraction of oxygen; PaCO_2_: partial pressure of carbon dioxide; PaO_2_: partial pressure of oxygen; PEEP: positive end-expiratory pressure.

*: *p* < 0.050 *vs* Mild-Moderate ARDS; °: *p* < 0.050 *vs* Fixed PEEP.

|  | Low Recruitability  4 ± 1  n = 13 | High Recruitability  27 ± 7  n = 22 | *p_RECRUIT_* | *p_METHOD_* | *p_INTER_* |
| --- | --- | --- | --- | --- | --- |
| PEEP, *cmH_2_O*  Clinical PEEP  Empirical PEEP | 10 [8 – 10]  16 [10 – 18] | 10 [8 – 10]  15 [10 – 18] | *-* | *-* | *-* |
| Peak pressure, *cmH_2_O*  Clinical PEEP  Empirical PEEP | 30 ± 9  37 ± 9 | 30 ± 6  36 ± 7 | *0.799* | ***<0.001*** | *0.956* |
| End-inspiratory airway pressure, *cmH_2_O*  Clinical PEEP  Empirical PEEP | 21 ± 4  28 ± 8 | 21 ± 4  27 ± 6 | *0.950* | ***<0.001*** | *0.439* |
| Driving pressure, *cmH_2_O*  Clinical PEEP  Empirical PEEP | 12 ± 2  14 ± 4 | 12 ± 3  13 ± 3 | *0.612* | ***0.003*** | *0.401* |
| E_RS_, *cmH_2_O/L*  Clinical PEEP  Empirical PEEP | 25 ± 6  28 ± 10 | 25 ± 8  26 ± 8 | *0.703* | ***0.008*** | *0.293* |
| C_RS_, *mL/cmH_2_O*  Clinical PEEP  Empirical PEEP | 43 ± 12  40 ± 16 | 45 ± 18  42 ± 13 | *0.692* | *0.130* | *0.915* |
| E_L_, *mL/cmH_2_O*  Clinical PEEP  Empirical PEEP | 20 ± 6  24 ± 10 | 18 ± 7  20 ± 7 | *0.269* | ***0.004*** | *0.253* |
| C_L_, *cmH_2_O/L*  Clinical PEEP  Empirical PEEP | 55 ± 20  50 ± 24 | 65 ± 28  57 ± 22 | *0.314* | *0.084* | *0.867* |
| End-expiratory transpulmonary pressure, *cmH_2_O/L*  Clinical PEEP  Empirical PEEP | -2.7 ± 4.7  1.3 ± 5.0 | -4.3 ± 5.1  -1.2 ± 6.0 | *0.248* | ***<0.001*** | *0.560* |
| End-inspiratory transpulmonary pressure, *cmH_2_O/L*  Fixed PEEP  Empirical PEEP | 7.2 ± 5.1  13.2 ± 7.5 | 4.6 ± 4.8  8.8 ± 4.9 | *0.056* | ***<0.001*** | *0.328* |
| Lung stress, *cmH_2_O*  Fixed PEEP  Empirical PEEP | 17 ± 4  23 ± 8 | 15 ± 4  20 ± 6 | *0.299* | ***<0.001*** | *0.252* |
| Mechanical Power, *J/min*  Fixed PEEP  Empirical PEEP | 20.0 ± 7.7  24.9 ± 8.3 | 18.3 ± 7.4  23.9 ± 8.0 | *0.622* | ***<0.001*** | *0.733* |
| Arterial PO_2_, *mmHg*  Fixed PEEP  Empirical PEEP | 82 ± 18  78 ± 19 | 76 ± 21  90 ± 35 | *0.667* | *0.433* | *0.164* |
| Arterial PCO_2_, *mmHg*  Fixed PEEP  Empirical PEEP | 45 ± 5  51 ± 8° | 46 ± 7  47 ± 8 | *0.496* | ***0.003*** | ***0.010*** |
| Ventilatory Ratio  Fixed PEEP  Empirical PEEP | 1.5 ± 0.3  1.7 ± 0.4 | 1.4 ± 0.3  1.6 ± 0.4 | *0.263* | ***0.001*** | *0.376* |

*Bibliography*

1. Chiumello D, Consonni D, Coppola S, Froio S, Crimella F, Colombo A. The occlusion tests and end-expiratory esophageal pressure: measurements and comparison in controlled and assisted ventilation. Ann Intensive Care 2016;6:13.

2. Herrmann P, Nguyen X, Luecke T. Del S zur analyse computertomographischer S. Virtuelle instrumente in der praxis Heidelberg: Huethig Verlag 2002:389–95.

3. Gattinoni L, Caironi P, Cressoni M, Chiumello D, Ranieri VM, Quintel M, Russo S, Patroniti N, Cornejo R, Bugedo G. Lung Recruitment in Patients with the Acute Respiratory Distress Syndrome. New England Journal of Medicine 2006;354:1775–86.

4. Gattinoni L, Chiumello D, Carlesso E, Valenza F. Bench-to-bedside review: chest wall elastance in acute lung injury/acute respiratory distress syndrome patients. Crit Care 2004;8:350.

5. Chiumello D, Bonifazi M, Pozzi T, Formenti P, Papa GFS, Zuanetti G, Coppola S. Positive end-expiratory pressure in COVID-19 acute respiratory distress syndrome: the heterogeneous effects. Crit Care 2021;25.

6. Chiumello D, Gotti M, Guanziroli M, Formenti P, Umbrello M, Pasticci I, Mistraletti G, Busana M. Bedside calculation of mechanical power during volume- and pressure-controlled mechanical ventilation. Crit Care 2020;24:417.

7. Gattinoni L, Tonetti T, Cressoni M, Cadringher P, Herrmann P, Moerer O, Protti A, Gotti M, Chiurazzi C, Carlesso E, Chiumello D, Quintel M. Ventilator-related causes of lung injury: the mechanical power. Intensive Care Med 2016;42:1567–75.

8. Sinha P, Calfee CS, Beitler JR, Soni N, Ho K, Matthay MA, Kallet RH. Physiologic Analysis and Clinical Performance of the Ventilatory Ratio in Acute Respiratory Distress Syndrome. Am J Respir Crit Care Med 2019;199:333–41.

9. Beitler JR, Sarge T, Banner-Goodspeed VM, Gong MN, Cook D, Novack V, Loring SH, Talmor D. Effect of Titrating Positive End-Expiratory Pressure (PEEP) With an Esophageal Pressure–Guided Strategy vs an Empirical High PEEP-FiO2 Strategy on Death and Days Free From Mechanical Ventilation Among Patients With Acute Respiratory Distress Syndrome. JAMA 2019;321:846.
